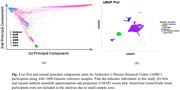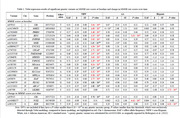# Genetic Association Analyses of Longitudinal Cognitive Changes Related to Alzheimer’s Disease in Diverse Populations

**DOI:** 10.1002/alz.095497

**Published:** 2025-01-09

**Authors:** Xian Wu, Khine Zin Aung, Erin L. Abner, Peter T Nelson, David W. Fardo, Yuriko Katsumata

**Affiliations:** ^1^ Sanders‐Brown Center on Aging, University of Kentucky, Lexington, KY USA; ^2^ College of Public Health, University of Kentucky, Lexington, KY USA; ^3^ College of Medicine, University of Kentucky, Lexington, KY USA

## Abstract

**Background:**

Late‐onset Alzheimer’s disease (LOAD) is highly heritable. ^1^ Recent research suggested that population‐specific LOAD genetic risks may exist. ^2^ The Mini‐Mental State Examination (MMSE; a measure of global cognitive function) has been commonly used to monitor AD‐related cognitive changes. MMSE raw scores have a strong ceiling effect (upper limit = 30 points). In a previous study, we demonstrated that the Tobit model utilizing ceiling information for estimation, is a superior approach compared to the linear model. ^3^ In this study, we aim to employ Tobit modeling to investigate associations of single nucleotide variants (SNVs) with MMSE scores to identify population‐specific genetic risks.

**Method:**

The phenotype data were drawn from the National Alzheimer’s Coordinating Center (NACC) Uniform Data Set (UDS) September 2022 data freeze. The genotype data were obtained from the Alzheimer’s Disease Genetics Consortium (ADGC). Alongside self‐reported race/ethnicity, we applied the principal component analysis (PCA) and uniform manifold approximation and projection (UMAP) to identify genetic‐ancestry (GA) groups (Fig. 1). Within each GA group, we performed association analyses of 82 AD‐related SNVs identified by a genome‐wide association study (Bellenguez et al., 2022), on MMSE using mixed‐effect Tobit modeling, adjusting for age at baseline, sex, education, and 2‐4 PC scores. We utilized Bonferroni correction to set *P* = 6.10 × 10^−4^ for multiple testing.

**Result:**

Three GA groups were included in analyses: non‐Hispanic White (NHW, n = 15,112), African American (AA, n = 2,392), and Hispanic (n = 1,159). Genetic variants were associated with MMSE shown in Table 1. At baseline, in NHW, genes *CR1, SORT1, PRKD3, BIN1, INPPSD, MME, UNC5CL, CD2AP, TMEM106B, SPDYE, USP6NL, MS4A4A, EED, SLC24A4, SPPL2A, MAF, PLCG2, and ABCA7* were associated with initial cognitive performance (ICP). In contrast, only *BIN1* showed an association with ICP in AA, while *LILRB2* was associated with ICP in Hispanics. Moreover*, CR1* in NHW, *NCK2* in AA, and *EED* in Hispanics were associated with change in MMSE scores over time.

**Conclusion:**

Our findings underscore the importance of considering population‐specific genetic variants when studying AD‐related longitudinal cognitive changes. Novel genetic risks may vary across GA groups and be further revealed in future studies.